# Systematic Review and Meta‐Analysis of Secondary Treatment Failure and Immunogenicity with Botulinum Neurotoxin A in Multiple Indications—Comment on Recommending IncobotulinumtoxinA as First‐Line to Prevent Secondary Treatment Failure [Letter]

**DOI:** 10.1111/ene.70581

**Published:** 2026-03-31

**Authors:** Fabiano Nadson Magacho‐Vieira

**Affiliations:** ^1^ Department of Clinical, Aesthetic and Surgical Dermatology Batista Memorial Hospital, Rua Professor Dias Da Rocha Fortaleza Ceara Brazil; ^2^ Magacho Institute for Health Education Fortaleza Ceara Brazil

To the Editor,

I read with interest the systematic review and meta‐analysis by Walter et al. [1] on secondary treatment failure (STF) and neutralizing antibodies (NAbs) formation with botulinum neurotoxin type A (BoNT‐A). The authors provide a valuable compilation of the available literature. However, I am concerned that the concluding recommendation favoring incobotulinumtoxinA as a first‐line strategy to prevent these immunogenicity‐related outcomes may go beyond what the current evidence can robustly support.

Three features of the included evidence may limit cross‐product inference.

1. **Follow‐up duration is markedly unbalanced across formulations**. The reported absence of immunogenic failure with incobotulinumtoxinA in the included evidence is largely driven by relatively short follow‐up, frequently based on open‐label extension periods, such as the 36 week study by Marciniak et al. [2] In contrast, STF rates for abobotulinumtoxinA and onabotulinumtoxinA are often derived from long‐term observational cohorts with follow‐up measured in years to decades, such as Jochim et al. [3], who reported up to 27 years of observation for abobotulinumtoxinA and 17 years for onabotulinumtoxinA. Because immunogenic risk is cumulative with lifetime exposure, comparing short‐term extensions with multi‐decade clinical practice cohorts risks systematic bias.
2. **Open‐label extension cohorts may be responder‐enriched**. Entry into extension phases typically requires completion of the main study period and continued clinical need for reinjection. This design may under‐represent early discontinuation and early non‐response, thereby lowering the observed STF rate in long‐term extension datasets.
3. **Definitions of STF are heterogeneous**. Walter et al. [1] acknowledge that STF definitions varied considerably or were not defined in some original manuscripts. Pooling objective criteria, such as systematic worsening of validated severity scores (e.g., TSUI‐based criteria as used by Hefter et al. [4]), together with subjective outcomes (patient satisfaction or clinician‐assessed reinjection need) may reduce comparability across toxins and compromise the precision of pooled estimates.

Importantly, clinical failure is multifactorial. As Walter et al. [1] acknowledge, there is a complex and non‐linear relationship between neutralizing antibodies and clinical failure, with a substantial proportion of partial secondary failure occurring without detectable antibodies, consistent with prior meta‐analytic evidence [5]. Technical factors (muscle selection, dosing, injection interval, use of guidance, and disease progression) remain key determinants of apparent loss of response. Therefore, while differences in antigenic load may be plausible, the current evidence does not justify a definitive clinical hierarchy.

Clinical practice should therefore continue to prioritize optimized technique, appropriate dosing, and individualized injection intervals rather than endorsing a specific formulation as a first‐line strategy, while awaiting independent, long‐term, head‐to‐head studies using harmonized STF definitions.

## Author Contributions

**Fabiano Nadson Magacho‐Vieira:** conceptualization, investigation, writing – original draft, writing – review and editing.

## Ethics Statement

No photos or patient personal information are in this manuscript. No institutional approval was required for this publication. The author confirms that this manuscript adheres to the ethical policies of the journal, as noted on the journal's author guidelines page.

## Conflicts of Interest

Dr. F.N. Magacho‐Vieira is the medical director of Dermadream Corporation Brazil. The author reports no other conflicts of interest in this communication.

## Data Availability

Data sharing not applicable to this article as no datasets were generated or analysed during the current study.
